# Supplementary material for: The Influence of Selected Meteorological Factors on the Prevalence and Course of Stroke
Source: Medicina (Kaunas). 2021 Nov 8;57(11):1216. doi: 10.3390/medicina57111216 (PMC8619234; doi:10.3390/medicina57111216)
Supplement: Supplementary file 1 [file medicina-57-01216-s001.zip › medicina-1370781-supplementary.pdf]

Supplement

Table S1. Meteorological parameters and the neurological status of patients as per the NIHSS on the day of onset.

**Parameter**	**NIHSS (1)**<br>**0-4**<br>**N=262**	**NIHSS (2)**<br>**5-12**<br>**N=97**	**NIHSS (3)**<br>**13-42**<br>**N=43**	**P 1–2**	**P 1–3**	**P 2–3**	**p**
Mean temperature on the first day of stroke [°C]	8.85 ± 8.10<br>Median 7.3<br>[-7.2–27]	9.58 ± 7.76<br>Median 8.8<br>[-7.2–27]	7.74 ± 7.12<br>Median 5.6<br>[-3–23.8]	0.419	0.442	0.205	0.587
Mean temperature on the day preceding stroke [°C]	8.98 ± 7.83<br>Median 7.45<br>[-7.2–27]	9.47 ± 7.27<br>Median 9.3<br>[-4.5–26]	8.13 ± 7.25<br>Median 6.4<br>[-3.1–23.8]	0.631	0.586	0.368	0.103
Mean atmospheric pressure on the first day of stroke [hPa]	984.6 ± 8.6<br>Median 984.9<br>[946.9–1004.7]	985.6 ± 8.4<br>Median 986.5<br>[946.9–1000.2]	986.3 ± 7.9<br>Median 985.7<br>[969.5–1001.6]	0.353	0.220	0.611	0.979
Mean atmospheric pressure on the day preceding stroke [hPa]	984.8 ± 7.7<br>Median 984.6<br>[958.4–1004.7]	985.9 ± 7.7<br>Median 986<br>[963.5–1004.4]	985.7 ± 8.1<br>Median 984.7<br>[963.5–1001.8]	0.261	0.503	0.903	0.973
Mean relative humidity on the first day of stroke [%]	73.6 ± 13.2<br>Median 74.45<br>[43.2–96.4]	74.1 ± 13.5<br>Median 75<br>[45.2–95.8]	76.5 ± 12.4<br>Median 77.8<br>[43.6–95.6]	0.261	0.170	0.310	0.857
Mean wind speed on the first day of stroke [km/h]	9.8 ± 4.9<br>Median 8.2<br>[1.8–24.1]	8.8 ± 4.7<br>Median 7.4<br>[1.8–22.2]	8.7 ± 3.9<br>Median 8.1<br>[1.8–19.8]	0.111	0.412	0.683	0.461

NIHSS- National Institute of Health Stroke Scale

Table S2. Selected meteorological parameters on the day of stroke onset and types of stroke.

**Parameter**	**Hemorrhagic stroke**<br>**N=38**	**Ischaemic stroke**<br>**N=364**	**p**
Maximum temperature [°C]	14.2 ± 9.3<br>Median 13.25<br>Range [1.4–32.7]	13.7 ± 9.4<br>Median 12.6<br>Range [(-3.9)–34.7]	0.748

Minimum temperature [°C]	4.3 ± 6.4<br>Median 3.5<br>Range [(-7.2)–17.7]	4.1 ± 6.9<br>Median 2.7<br>Range [(-11.4)–21.0]	0.867
Mean temperature [°C]	9.1 ± 7.7<br>Median 7.3<br>Range [(-2.1)–23.3]	8.9 ± 7.9<br>Median 7.4<br>Range [(-7.2)–27]	0.836
Mean temperature on the day preceding stroke [°C]	9.2 ± 7.3<br>Median 6.7<br>Range [(-2.2)–23.3]	9.0 ± 7.7<br>Median 7.8<br>Range [(-7.2)–27]	0.945
Mean monthly temperature [°C]	9.4 ± 7.1<br>Median 7.6<br>Range [0.6–21.8]	9.0 ± 7.0<br>Median 7.6<br>Range [0.6–21.8]	0.618
Humidity [%]	77.0 ± 12.0<br>Median 79.15<br>Range [47.1–95.8]	73.7 ± 13.3<br>Median 74.4<br>Range [43.2–96.4]	0.140
Atmospheric pressure [hPa]	985.4 ± 8.6<br>Median 986.4<br>Range [946.9–997.9]	985.0 ± 8.5<br>Median 985.1<br>Range [946.9–1004.7]	0.794
Atmospheric pressure on the day preceding stroke [hPa]	984.6 ± 7.0<br>Median 983.9<br>Range [968.2–1001.8]	985.2 ± 7.8<br>Median 985.0<br>Range [958.4–1004.7]	0.637
Atmospheric pressure, monthly mean [hPa]	985.0 ± 3.1<br>Median 984.2<br>Range [981.4–994.2]	985.2 ± 3.4<br>Median 984.2<br>Range [981.4–994.2]	0.824
Wind speed [km/h]	8.24 ± 3.90<br>Median 7.3<br>Range [2.9–20.6]	9.55 ± 4.87<br>Median 8.1<br>Range [1.8–24.1]	0.109
Precipitation [mm]	1.22 ± 2.43<br>Median 0.2<br>Range [0–12]	1.53 ± 2.96<br>Median 0<br>Range [0–20.1]	0.540
Insolation [h]	4.78 ± 4.75<br>Median 3.95<br>Range [0–14.4]	4.54 ± 4.38<br>Median 3.3<br>Range [0–14.7]	0.683
